# Association and variation in performance, nutrient digestibility, carcass traits, and intestinal development of broiler chickens by various male-to-female ratios^✰^

**DOI:** 10.1016/j.vas.2026.100628

**Published:** 2026-03-23

**Authors:** Motaleb Ebrahimi, Mohsen Daneshyar, Sina Payvastegan, Hamed Ahmadi

**Affiliations:** a Department of Animal Science, Faculty of Agriculture, Urmia University, Urmia, Iran; b Institute of Animal Science, University of Hohenheim, Stuttgart, Germany

**Keywords:** Sex effect, Broiler chickens, Digestibility, Body weight, Correlation coefficients

## Abstract

Despite well-documented differences in growth performance and physiology between male and female broilers, incorporating varying male-to-female ratios into research designs offers a more industry-relevant framework, enhancing the applicability and translational value of experimental findings. The objective of this study was to determine the relationship and accurate estimate changes of performance, nutrient digestibility, carcass characteristics, coefficient of variation (**CV**) of performance, gut weight and length, and intestinal morphological parameters due to the use of different male-to-female ratios. Birds (*n* = 550) were separated by sex and placed in 11 groups (treatments) according to the male-female ratios: G100 (all males), G90 (9 males + 1 female), G80 (8 males + 2 females), G70 (7 males + 3 females), G60 (6 males + 4 females), G50 (5 males + 5 females), G40 (4 males + 6 females), G30 (3 males + 7 females), G20 (2 males + 8 females), G10 (1 male + 9 females) and G0 (all females). Body weight gain (**BWG**) showed a significant relationship with sex ratio, with the strongest association observed during the grower period (R² = 0.47; *P* = 0.006), followed by the finisher (R² = 0.27; *P* = 0.046), while no significant relationship was detected during the starter phase. Shifting the sex ratio from all-male to all-female reduced total feed intake and BWG by 6.94% and 10.98%, respectively, and increased feed conversion ratio by 4.53%. Single-sex rearing reduced the CV of BWG compared with mixed-sex groups. Apparent metabolizable energy (**AME**), crude protein, ether extract, and crude fiber digestibility decreased by 5.33%, 6.16%, 3.39%, and 20.75%, respectively, as the proportion of females increased. Pearson correlation analysis showed that BW was most strongly correlated with crude fiber digestibility (*r* = 0.90), small intestine weight (*r* = 0.88), villus height (*r* = 0.87), digestibility of AME (*r* = 0.87), ether extract (*r* = 0.82), and crude protein (*r* = 0.71). In conclusion, altering the male-to-female ratio resulted in distinct and quantifiable changes in performance, nutrient utilization, and intestinal development. These findings highlight that estimating sex ratio effects may enhance the alignment of academic research with commercial production conditions without requiring complete sex segregation.

## Introduction

In the poultry industry, evidence indicates that male broiler chickens exhibit faster growth rates and superior feed conversion efficiency compared to females (Ban et al., 2025; England et al., 2023). This performance advantage may result from a combination of physiological, genetic, and environmental factors. From a genetic perspective, heritable differences in growth rate and carcass composition between sexes have been reported (Livingston et al., 2020). Embryonic studies have further shown that early growth in male embryos may be influenced by more active metabolic pathways and a greater capacity for protein synthesis (Henry & Burke, 1998). Variations in the secretion and concentration of sex hormones, particularly androgens, may also stimulate anabolic pathways, thereby enhancing growth rate (Younis & Ghoneim, 2022). Moreover, the structure and composition of the gastrointestinal microbiota in males may differ from that of females, contributing to improved nutrient digestibility and absorption (Ahmadzadeh et al., 2025; England et al., 2024). Other contributing factors, such as differences in feed intake patterns, immune system efficiency, and responses to environmental stressors, may also partially explain the observed performance gap (Mahrous et al., 2023).

In research trials, to eliminate or control the effects of sex-related differences on productive, physiological, and behavioral parameters, single-sex flocks (all-male or all-female) or mixed-sex groups with fixed ratios (e.g., 50% male and 50% female) are often used (Gous, 2018). However, implementing these approaches in practice presents several challenges. In modern broiler strains, sex determination (sexing) at one day of age is difficult and requires specialized skills, with a notable error rate that can affect the accuracy of experimental results (England et al., 2021). Additionally, many hatcheries, due to biosecurity concerns, do not permit researchers to access chicks for sex determination. The sexing process itself can induce stress in day-old chicks and has been reported to increase mortality by up to 1% (Phelps et al., 2003). More importantly, the use of single-sex flocks may result in nutritional and management requirements that do not fully align with commercial industry conditions, which typically involve variable male-to-female ratios. This mismatch can limit the applicability and generalizability of research findings to real-world broiler production systems (England et al., 2023).

A novel approach to overcoming the limitations of sex separation in research is to design experimental treatments that reflect industry-representative sex ratios. In this method, without physically separating birds at hatch, different male-to-female ratios can be precisely incorporated into treatments, and with sufficiently large sample sizes, the effect of sex on all measured parameters can be accurately estimated. In other words, instead of using all-male or all-female groups, treatments should be designed with variable sex ratios that closely match those found in commercial farms. This approach not only simulates real-world production conditions but also improves research accuracy, reduces errors, and facilitates experimental implementation. Ultimately, this enables precise quantification of the true contribution of sex to variability in each parameter, providing reliable and practically relevant information without the need for hatch-day sexing and its associated drawbacks.

Studies have investigated the functional and physiological differences between male and female chickens (Ahmadzadeh et al., 2025; Ban et al., 2025) but these do not necessarily reflect the conditions found in commercial farms. On the other hand, a comprehensive and simultaneous study of the effect of different male-to-female ratios on performance, nutrient digestibility, carcass characteristics, coefficient of variation (**CV**) of performance, gut weight and length, and morphological parameters has not yet been fully conducted. This knowledge gap may result in industry decisions based on incomplete or non-generalizable information. Therefore, this study aimed to assess the impact of different male-to-female ratios on broiler performance and to generate results relevant for both research and commercial applications. It was hypothesized that altering the male-to-female ratio would result in distinct and measurable changes in growth performance, nutrient digestibility, carcass traits, and intestinal development. Furthermore, quantifying these changes would enable the use of such data for the development of predictive models, thereby eliminating the need for complete sex segregation in research trials and enhancing the generalizability and industry relevance of experimental findings.

## Materials and methods

This study was approved by the Animal Care Committee and Animal Research Ethics Board of Urmia University, Urmia, Iran (Approval No:IR-UU-AEC-3/76).

### Birds, treatments, and management

A total of 550 male and female Ross 308 one-day-old chicks (body weight (**BW**) mean of 45.7 ± 0.28 g) were used in a completely randomized design with 11 treatments, 5 replicates, and 10 chicks per replicate. Upon arrival, all birds were weighed, vent sexed (genital sex determination), and allocated to 55 floor pens. The experiment lasted for 42 d Birds (*n* = 550) were separated by sex and placed in 11 groups (pens) according to the male-female ratios: G100 (all males), G90 (9 males+ 1 female), G80 (8 males+ 2 females), G70 (7 males+ 3 females), G60 (6 males+ 4 females), G50 (5 males+ 5 females), G40 (4 males+ 6 females), G30 (3 males+ 7 females), G20 (2 males+ 8 females), G10 (1 male+ 9 females) and G0 (all females). The birds were housed in floor cages measuring 100 cm length × 70 cm width × 60 cm height. The lighting program was maintained at 23L:1D for the first 7 days, followed by 18L:6D for the rest of the experimental period. Relative humidity was maintained at approximately 50% throughout the experimental period. Ambient temperature was adjusted according to broiler age based on the Ross 308 management recommendations (Ross 308 Broiler Management Handbook 2022 EN – Aviagen). The Temperature–Humidity Index (THI) was calculated using the following equation (Silva et al., 2025).

**Fig. 1:**
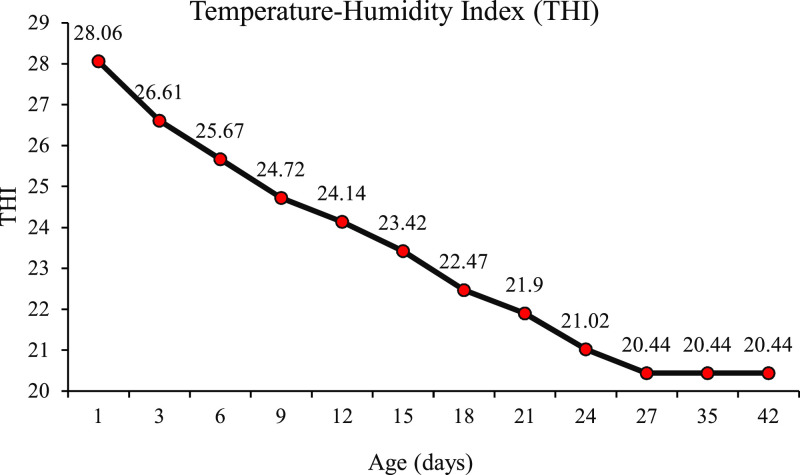
Temperature–Humidity Index (THI) during the experimental period.

THI=T−(0.55−0.0055×RH)×(T−14.5)

where T represents ambient temperature (°C) and RH represents relative humidity. A graphical representation of THI changes across age is presented in Fig. 1. All groups were fed the same diets during starter (1–10d), grower (11–24d), and finisher (25–42d) periods (Table 1). The birds had free access to feed and water. Vaccination against Newcastle disease was conducted via drinking water on days 7, 17, and 28, and against infectious bursal disease on day 14.

**Table 1:** Components and chemical compositions of the diets during starter, growth and finisher periods.

Diet Ingredients (%)	Starter (d 1–10)	Grower (d 11–24)	Finisher (d 25–42)
Corn	55.97	59.97	66.35
Soybean meal 44%	37.58	34.16	28.43
Soybean oil	1.60	1.57	1.53
Dicalcium phosphate	2.47	2.09	1.65
Calcium carbonate	0.68	0.57	0.46
L-Lys HCl	0.31	0.29	0.27
DL-Met	0.35	0.31	0.28
L-Thr	0.14	0.13	0.11
Sodium bicarbonate	0.10	0.12	0.15
Vitamin and mineral-Premix^1^	0.50	0.50	0.50
Salt	0.30	0.29	0.27
Sum	100	100	100
**Calculated composition**			
AMEn (Kcal/Kg)	2796	2843	2914
Crude protein (%)	21.62	20.37	18.33
Ether extract (%)	4.03	4.08	4.15
Crude fiber (%)	3.29	3.21	3.10
Linoleic acid (%)	2.20	2.27	2.35
Calcium (%)	0.89	0.77	0.61
Available phosphorus (%)	0.47	0.40	0.33
Sodium (%)	0.17	0.17	0.17
Digestible Methionine (%)	0.63	0.58	0.53
Digestible Methionine+ Cysteine (%)	0.94	0.88	0.80
Digestible Lysine (%)	1.24	1.14	1.01
Digestible Threonine (%)	0.82	0.75	0.67
Digestible Arginine (%)	1.23	1.15	1.01
Digestible Valine (%)	0.86	0.80	0.72
Digestible Isoleucine (%)	0.82	0.75	0.67
DCAB (mEq/kg)	231.76	217.69	199.31

Abbreviations: AMEn, apparent metabolizable energy corrected for nitrogen; DCAB, dietary cation–anion balance. ^1^Provides the following per kg of diet: 4.13 mg retinol, 60.00 µg chole-calciferol, 30.00 mg Dl-α-tocopherol, 3 mg menadione, 2.20 mg thiamine, 8.00 mg riboflavin, 5.00 mg pyridoxine, 11.00 µg cyanocobalamin, 1.50 mg folic acid, 150.00 µg biotin, 25.00 mg calcium pantotenate, 65.00 mg nicotinic acid. Provides the following per kg of diet: 60.00 mg Mn (manganese sulphate), 40.00 mg Zn (zinc oxide), 0.33 mg I (potassium iodate), 80.00 mg Fe (ferrous sulphate), 8.00 mg Cu (copper sulphate), 0.15 mg Se (sodium selenite), 150.00 mg ethoxyquin.

### Performance

To calculate the amount of feed intake (**FI**) of each repetition, the amount of feed remaining at the end of each period was deducted from the total feed given during the period. On days 1, 10, 24, and 42, all chickens of each experimental unit were weighed as a group. To calculate the body weight gain (**BWG**) of each repetition in each period, the difference between the final weight and the beginning of the breeding period was determined. The feed conversion ratio (**FCR**) was also calculated by dividing the average FI by the average BWG of chickens for each period (Gouran et a., 2025).

### Coefficient of variation (%) of performance

The BW, FI, and FCR per pen within each treatment group were calculated to determine the CV of the performance parameters according to the following equation:

$$\text{CV}\%=\left(SD_{p}/{\bar{x}}_{p}\right)\times 100.$$

where SD_p_ is the standard deviation of BWG, FI, or FCR within a treatment group; and x̄_p_ represents the mean values of the performance parameters.

### Digestibility assay

To determine apparent ileal digestibility coefficients of nutrients, 3 day before ileal digesta collection (Day 39), 5 g of titanium dioxide per kg feed was added to the diet as an exogenous marker (Nari & Ghasemi, 2020). At the end of the experiment (on Day 42), all the birds were euthanized by CO_2_ asphyxiation and the contents from the distal portion of the ileum, spanning from Meckel's diverticulum to the ileo-cecal-colonic junction, were obtained by flushing with distilled water into plastic containers. The diets and ileal contents were oven-dried (60°C for 72 h) and then left in the open air for 24 h to equilibrate. The diets and ileal contents were ground (<0.75 mm) and the dry matter (**DM**), crude protein (**CP**), ether extract (**EE**), and crude fiber (**CF**) contents were measured according to standard methods (AOAC, 2006). The gross energy (**GE**) of the experimental diets and ileal contents was determined using a bomb calorimeter (Parr 6200 bomb calorimeter, Parr Instruments Co., Moline, IL) with benzoic acid as the calibration standard. The optical absorbance of the samples was measured by a spectrophotometer (Spectronic 21D; Milton Roy Co., Rochester, NY, USA) at a wavelength of 440 nm and the amount of titanium oxide in the samples was determined by comparing it with a standard curve (Short et al., 1996).

Apparent total-tract nutrient digestibilities were calculated by the index method, which follows the equation described by Kong and Adeola (2014):

Apparent nutrient digestibility (AND, %) = [1 – (T_i_ ÷ t_o_) × (x_o_ ÷ x_i_)] × 100

Where AND is the apparent total tract digestibility of DM, CP, and EE expressed in percentage; T_i_ represents the concentration of titanium (g/kg DM) in experimental diets, and T_o_ represents the concentration of titanium (g/kg DM) in excreta output; X_i_ and X_o_ are the concentration of nutrients in experimental diets and excreta output, respectively.

The apparent metabolizable energy (**AME**) was calculated using the following equations (Rahbari et al., 2025):

AME (kcal/kg) = GE diet – (GE feces × Titanium dioxide diet / Titanium dioxide feces)

### Carcass analysis

At day 42 of age, all birds were euthanized by CO_2_ asphyxiation after approximately 4 h of feed withdrawal. Before euthanized, the birds selected for dissection were weighed again on a digital scale. Viscera were removed immediately, and the weights of carcass, breast, leg, abdominal fat, duodenum, jejunum, and ileum were measured using a digital scale after that (0.01 g; KEB 602, China). All of the data regarding carcass parts and internal organ weights were expressed as a percentage of live BW.

### Intestinal morphology

At 42 days of age, the jejunum of all birds from two replicates of each treatment (20 birds) was dissected for morphological evaluation and analyzed according to the method of Liu et al. (2022). Briefly, 1 cm of the jejunum was removed and washed with 0.9% saline to remove the contents. All samples were fixed in 10% buffered formalin for histological evaluation. Intestinal morphological measurements included villus height, villus width, and crypt depth in each section. The villus height-to-crypt depth (**VH/CD**) ratio was then estimated by dividing villus height by crypt depth (Delfani et al., 2022).

### Statistical analysis

The obtained data were tested for normality using the PROC UNIVARIATE of SAS, version 9.2, and whenever needed, the percentage data were normalized by ArcSin√x transformation. The data were analyzed using the PROC GLM of SAS (version 9.2). The difference among means was determined using Duncan's test, and P values <0.05 were considered statistically significant. The statistical model of the current study was as follows:

$$Y_{\text{ij}}=\mu +T_{i}+\varepsilon_{\text{ij}}$$

where Y_ij_, μ, T_i_, and ε_ij_ represent the observation, the mean of observations, the treatment effect, and the experimental error of each observation, respectively.

Graphical illustrations were performed using Microsoft Excel (Microsoft Office 365, Microsoft Corp., USA). Pearson’s correlation coefficient (r) was calculated to examine the linear relationships between the proportion of males in each treatment and the performance parameters, including nutrient digestibility, carcass traits, and BW. The goodness of fit of the models was assessed using the coefficient of determination (R²), and the results were presented as scatter plots.

## Results

### Broiler performance

**Fig. 2:**
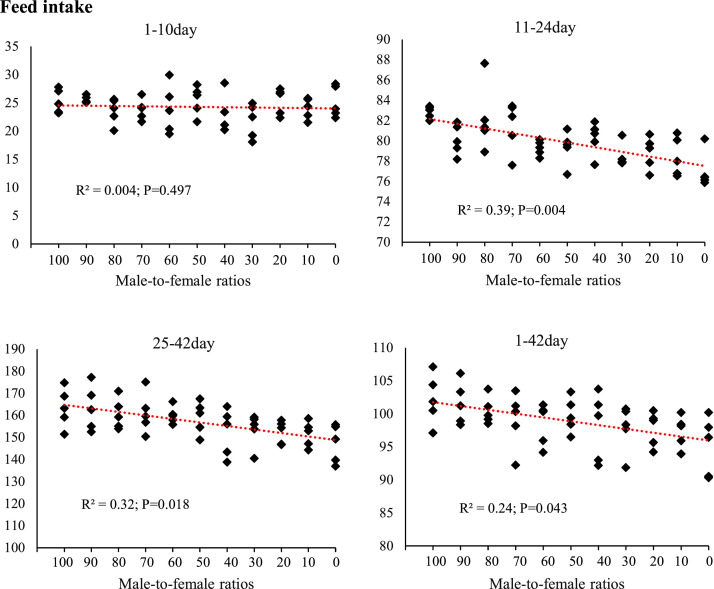
The effect of different male-to-female ratios of broiler chickens on feed intake (g/d) in the starter (1–10 d), grower (11–24 d) and finisher (25–42 d) periods. Experimental groups: G100 (all males), G90 (9 males+ 1 female), G80 (8 males+ 2 females), G70 (7 males+ 3 females), G60 (6 males+ 4 females), G50 (5 males+ 5 females), G40 (4 males+ 6 females), G30 (3 males+ 7 females), G20 (2 males+ 8 females), G10 (1 male+ 9 females) and G0 (all females). (*n* = 10).

**Fig. 3:**
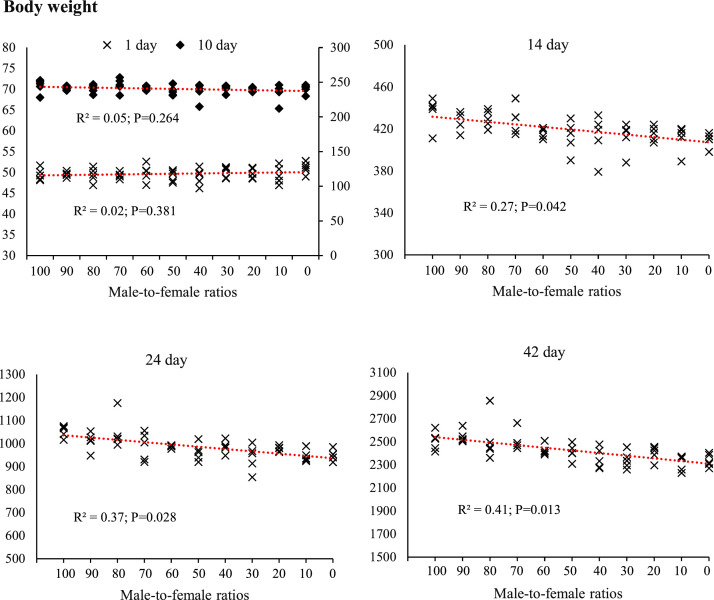
The effect of different male-to-female ratios of broiler chickens on body weight (g) in the starter (1–10 d), grower (11–24 d) and finisher (25–42 d) periods. Experimental groups: G100 (all males), G90 (9 males+ 1 female), G80 (8 males+ 2 females), G70 (7 males+ 3 females), G60 (6 males+ 4 females), G50 (5 males+ 5 females), G40 (4 males+ 6 females), G30 (3 males+ 7 females), G20 (2 males+ 8 females), G10 (1 male+ 9 females) and G0 (all females). (*n* = 10).

**Fig. 4:**
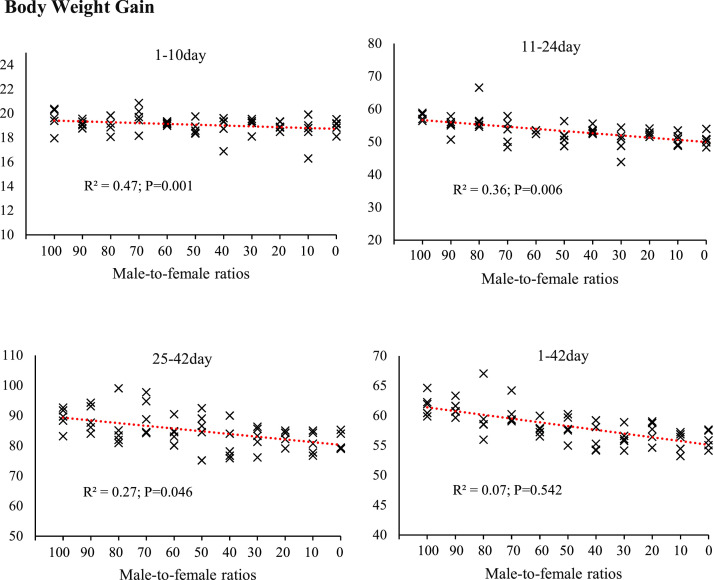
The effect of different male-to-female ratios of broiler chickens on body weight gain (g/d) in the starter (1–10 d), grower (11–24 d) and finisher (25–42 d) periods. Experimental groups: G100 (all males), G90 (9 males+ 1 female), G80 (8 males+ 2 females), G70 (7 males+ 3 females), G60 (6 males+ 4 females), G50 (5 males+ 5 females), G40 (4 males+ 6 females), G30 (3 males+ 7 females), G20 (2 males+ 8 females), G10 (1 male+ 9 females) and G0 (all females). (*n* = 10).

**Fig. 5:**
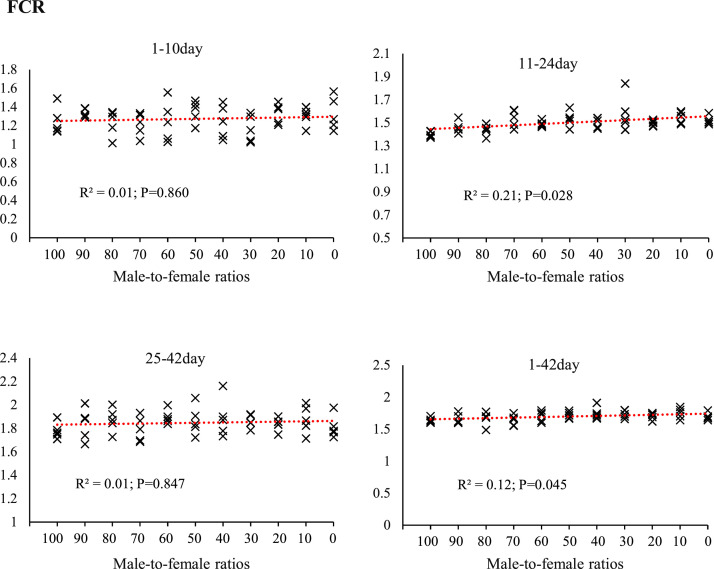
The effect of different male-to-female ratios of broiler chickens on feed conversion ratio in the starter (1–10 d), grower (11–24 d) and finisher (25–42 d) periods. Experimental groups: G100 (all males), G90 (9 males+ 1 female), G80 (8 males+ 2 females), G70 (7 males+ 3 females), G60 (6 males+ 4 females), G50 (5 males+ 5 females), G40 (4 males+ 6 females), G30 (3 males+ 7 females), G20 (2 males+ 8 females), G10 (1 male+ 9 females) and G0 (all females). (*n* = 10).

Fig. 2, Fig. 3, Fig. 4, Fig. 5 illustrates the effects of sex ratio on growth performance parameters, including FI, BW, BWG, and FCR across different rearing phases. During the starter period (1–10 days of age), variations in FI among the groups were minimal (R²= 0.004; *P* = 0.497), indicating a negligible impact of sex ratio in this phase. In contrast, greater differences were observed during the grower (11–24 days) and finisher (25–42 days) periods (R²= 0.35; *P* = 0.004 and R²= 0.32; *P* = 0.018, respectively), with groups containing a higher proportion of males tending to consume more feed. This trend was also evident over the entire rearing period (1–42 days), with an R² of 0.24. BW patterns at different ages indicated that from day 14 onwards, the slope of variation among groups became more noticeable (R²= 0.27; *P* = 0.042). At day 42, marking the end of the rearing period, the highest R² (R²= 0.41; *P* = 0.013) was recorded, with groups containing more males achieving greater BWs. BWG at different growth stages was correlated with sex ratio, with the strongest relationship in the grower period (R²= 0.36; *P* = 0.006), followed by the finisher (R²= 0.27; *P* = 0.046) and starter periods (R²= 0.07; *P* = 0.542). Between-group differences in FCR were smaller compared with other performance indicators. However, in the finisher period and over the entire rearing period (1–42 days), R² values of 0.21 and 0.12, respectively, were recorded, suggesting that sex ratio may moderately affect feed conversion efficiency in the later stages of rearing.

Table 2 shows the percentage changes in performance indices (FI, BWG, and FCR) in response to changes in sex ratio during different rearing periods. The values were calculated based on the percentage difference compared to the G100 (100% male). The results showed that by changing the male-female ratio from 100% male to 100% female, FI decreased by 6.947% for the total period. In the BWG index, changing the sex ratio from 100% male to 100% female caused a 10.985% decrease, and the largest decrease was also observed in this group. For FCR, this change in sex ratio caused a 4.536% increase (decrease in efficiency), while the largest increase (5.834%) was related to G10.

**Table 2:** The percentage changes in performance indices in response to changes in male-to-female ratio during different rearing periods.

Groups	Average feed intake (g/d)				Body weight gain (g/d)				=Feed conversion ratio (FCR)			
	Starter	Grower	Finisher	Total	Starter	Grower	Finisher	Total	Starter	Grower	Finisher	Total
G100	0.000	0.000	0.000	0.000	0.000	0.000	0.000	0.000	0.000	0.000	0.000	0.000
G90	+1.228	-3.284	-0.098	-0.597	-1.844	-4.848	-0.079	-0.987	+3.130	+1.643	-0.019	+0.394
G80	-6.696	-0.060	-1.725	-1.693	-1.947	-0.121	-3.401	-3.106	-4.843	+0.061	+1.735	+1.459
G70	-5.666	-1.678	-1.462	-3.043	+0.717	-8.102	+1.010	-2.249	-6.337	+6.991	-2.448	-0.812
G60	-5.309	-4.298	-2.086	-3.650	-1.844	-7.843	-5.085	-6.407	-3.530	+3.847	+3.159	+2.946
G50	+0.792	-4.226	-2.674	-2.358	-3.740	-10.284	-4.018	-6.132	+4.708	+6.753	+1.401	+4.020
G40	-7.726	-3.115	-6.798	-4.119	-3.842	-7.445	-9.159	-9.092	-4.039	+4.678	+2.600	+5.470
G30	-13.708	-5.264	-6.070	-4.295	-2.203	-13.608	-7.397	-8.947	-11.765	+9.659	+1.434	+5.108
G20	+0.317	-4.853	-6.761	-4.374	-2.766	-8.812	-6.903	-7.297	+3.171	+4.342	+0.153	+3.153
G10	-4.873	-5.324	-7.305	-4.765	-5.225	-12.136	-9.227	-10.015	+0.372	+7.753	+2.116	+5.834
G0	-0.357	-7.039	-9.863	-6.947	-7.531	-13.158	-9.833	-10.985	+7.758	+7.047	+0.033	+4.536

Experimental groups: G100 (all males), G90 (9 males+ 1 female), G80 (8 males+ 2 females), G70 (7 males+ 3 females), G60 (6 males+ 4 females), G50 (5 males+ 5 females), G40 (4 males+ 6 females), G30 (3 males+ 7 females), G20 (2 males+ 8 females), G10 (1 male+ 9 females) and G0 (all females).

### Coefficient of variation

**Fig. 6:**
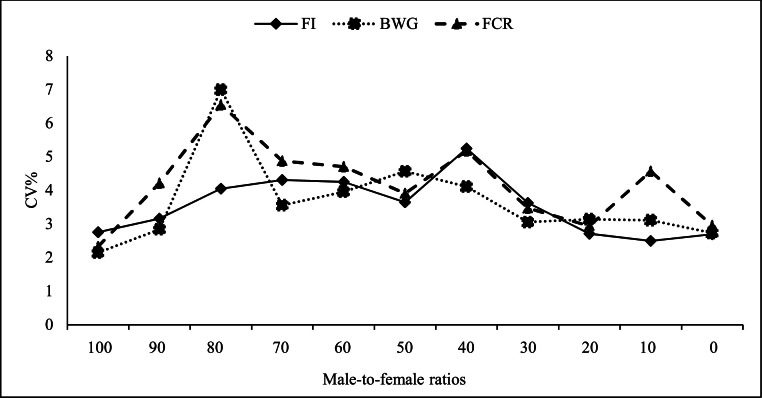
Coefficient of variation (CV%) of body weight gain per bird (BWG), feed intake per bird (FI), feed conversion ratio (FCR) of broiler chickens during whole period (1 to 42 days of age). Experimental groups: G100 (all males), G90 (9 males+ 1 female), G80 (8 males+ 2 females), G70 (7 males+ 3 females), G60 (6 males+ 4 females), G50 (5 males+ 5 females), G40 (4 males+ 6 females), G30 (3 males+ 7 females), G20 (2 males+ 8 females), G10 (1 male+ 9 females) and G0 (all females). (*n* = 50).

Fig. 6 shows the effect of gender composition on CV for FI, BWG and FCR during the whole period (1–42 days). CV of FI, BWG and FCR were greater in mixed-sex pens (especially pens with more males) than single-sex male and single-sex female pens. The CV level of BWG was also the highest in G80 (6.99%), decreased with the increase in the ratio of female to male, so that it reached its minimum in the single-sex female group (2.73%). Of course, CV was at a minimum level in single-sex male pens (2.14%) and 9 male broilers (2.83%).

### Nutrient digestibility

**Fig. 7:**
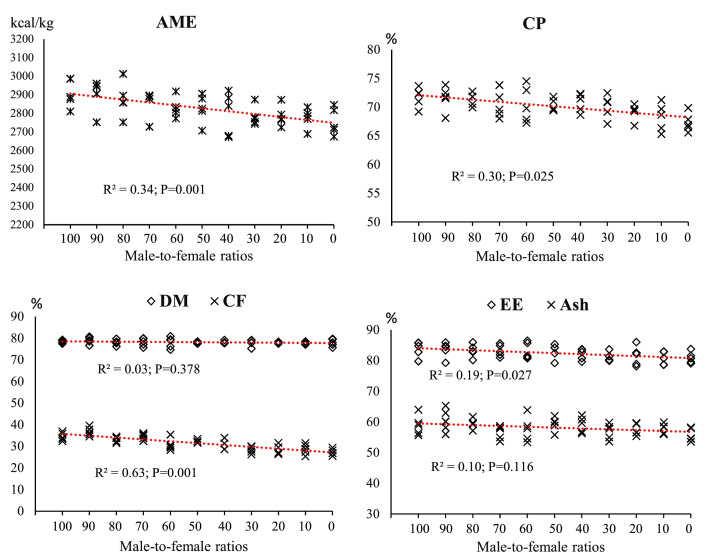
The effect of different male-to-female ratios of broiler chickens on AME and nutrient digestibility. Experimental groups: G100 (all males), G90 (9 males+ 1 female), G80 (8 males+ 2 females), G70 (7 males+ 3 females), G60 (6 males+ 4 females), G50 (5 males+ 5 females), G40 (4 males+ 6 females), G30 (3 males+ 7 females), G20 (2 males+ 8 females), G10 (1 male+ 9 females) and G0 (all females). (*n* = 10).

The effects of different male to female ratios on nutrient digestibility and AME are reported in Fig. 7. AME values showed a moderate negative correlation with changes in the sex composition of the groups (R²= 0.34; *P* = 0.001). As the proportion of female broilers increased, a gradual decrease in AME values was observed, indicating that male-dominated groups achieved higher levels of metabolizable energy. Digestibility of CF was the most correlated (R²= 0.63; *P* = 0.001) and decreased significantly with increasing female proportion. CP digestibility (R²= 0.30; *P* = 0.025) and EE (R²= 0.19; *P* = 0.027) also decreased with increasing female proportion, although the magnitude of this decrease was less.

Table 3 shows the percentage changes in nutrient digestibility and AME in response to changes in sex ratio. Values are calculated based on the percentage difference from group G100 (100% male). The results showed that as the male-female ratio changed from 100% male to 100% female, AME decreased by 5.34%, and the decreasing trend was almost intensified from group G70 onwards. In CP digestibility, a gradual decrease started in G90 (0.25%) and reached the largest decrease (6.16%) in the all-female group (G0). EE digestibility also showed the largest decrease in G10 (3.558%).

**Table 3:** The percentage changes in AME and nutrient digestibility in response to changes in male-to-female ratio.

Groups	AME (kcal/kg)	DM (%)	CP (%)	EE (%)	CF (%)	Ash (%)
G100	0.000	0.000	0.000	0.000	0.000	0.000
G90	-0.249	+0.611	-0.250	-0.226	-1.492	+2.681
G80	-1.172	-0.598	-0.501	-0.620	-3.797	+0.797
G70	-1.814	-0.382	-1.714	-0.620	+0.028	-3.835
G60	-2.764	-0.598	-1.756	-0.788	-10.811	-2.104
G50	-2.841	-0.623	-2.006	-0.847	-6.173	+0.627
G40	-3.792	-0.445	-1.254	-2.017	-10.521	+0.339
G30	-4.305	-0.878	-2.285	-2.925	-17.594	-3.428
G20	-4.268	-0.598	-3.623	-2.638	-17.913	-1.357
G10	-4.598	-0.993	-4.905	-3.558	-16.956	-1.917
G0	-5.338	-0.725	-6.160	-3.391	-20.753	-5.346

Abbreviations: AME: apparent metabolizable energy; DM: dry matter; CP: crude protein; EE: ether extract; CF: crude fiber.

Experimental groups: G100 (all males), G90 (9 males+ 1 female), G80 (8 males+ 2 females), G70 (7 males+ 3 females), G60 (6 males+ 4 females), G50 (5 males+ 5 females), G40 (4 males+ 6 females), G30 (3 males+ 7 females), G20 (2 males+ 8 females), G10 (1 male+ 9 females) and G0 (all females).

### Carcass characteristics

**Fig. 8:**
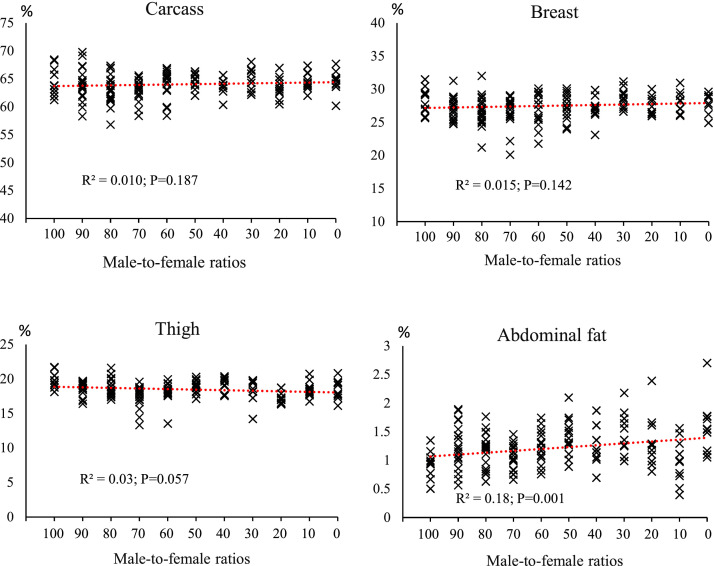
The effect of different male-to-female ratios of broiler chickens on carcass traits (% of body weight). Experimental groups: G100 (all males), G90 (9 males+ 1 female), G80 (8 males+ 2 females), G70 (7 males+ 3 females), G60 (6 males+ 4 females), G50 (5 males+ 5 females), G40 (4 males+ 6 females), G30 (3 males+ 7 females), G20 (2 males+ 8 females), G10 (1 male+ 9 females) and G0 (all females). (*n* = 20).

**Fig. 9:**
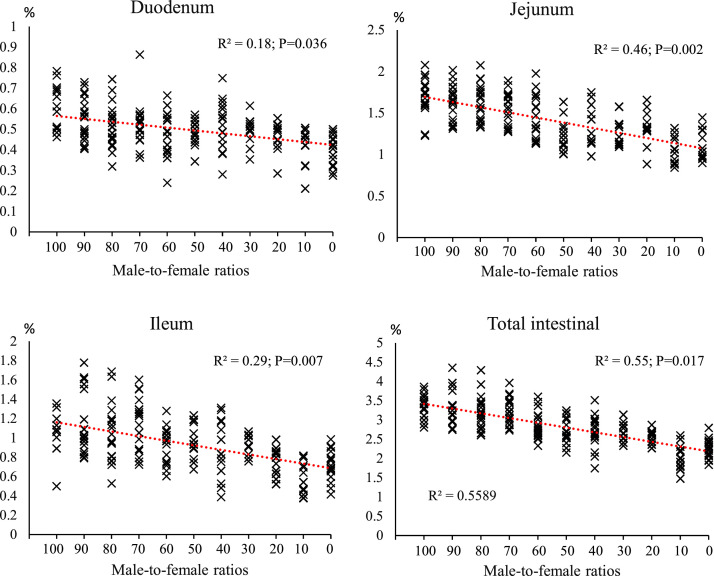
The effect of different male-to-female ratios of broiler chickens on weight of different parts of the small intestine (% of body weight). Experimental groups: G100 (all males), G90 (9 males+ 1 female), G80 (8 males+ 2 females), G70 (7 males+ 3 females), G60 (6 males+ 4 females), G50 (5 males+ 5 females), G40 (4 males+ 6 females), G30 (3 males+ 7 females), G20 (2 males+ 8 females), G10 (1 male+ 9 females) and G0 (all females). (*n* = 20).

**Fig. 10:**
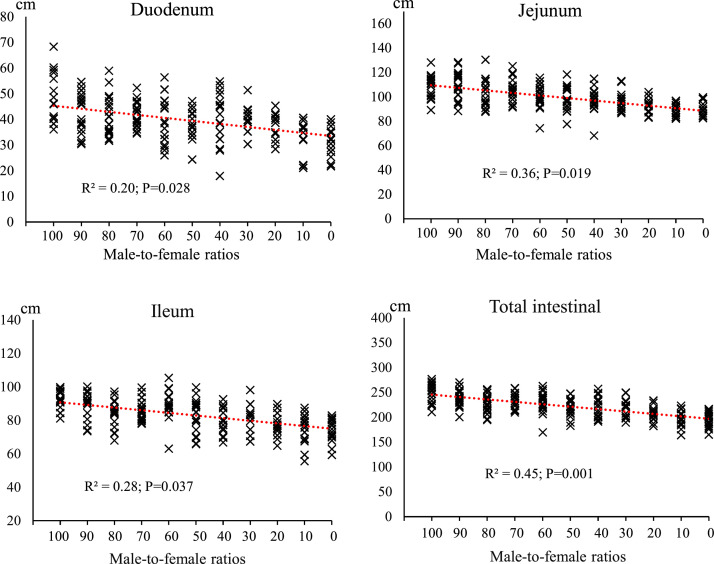
The effect of different male-to-female ratios of broiler chickens on length of different parts of the small intestine (cm). Experimental groups: G100 (all males), G90 (9 males+ 1 female), G80 (8 males+ 2 females), G70 (7 males+ 3 females), G60 (6 males+ 4 females), G50 (5 males+ 5 females), G40 (4 males+ 6 females), G30 (3 males+ 7 females), G20 (2 males+ 8 females), G10 (1 male+ 9 females) and G0 (all females). (*n* = 20).

Fig. 8, Fig. 9, Fig. 10 shows the effect of different male-to-female ratios on carcass characteristics and small intestinal segments. Among the experimental groups, changes in carcass, breast and thigh percentages were insignificant, as indicated by the low R² values (R²= 0.010; *P* = 0.187, R²= 0.015; *P* = 0.142 and R²= 0.03; *P* = 0.057, respectively). Abdominal fat accumulation showed moderate changes in response to the sex composition of the groups, with an R² value of 0.18 (*P* = 0.001). There was a noticeable increasing trend with increasing female proportion, indicating that female-dominated groups tend to store more fat. Intestinal weight was more sensitive to sex than carcass characteristics. The jejunum had the highest correlation (R²= 0.46; *P* = 0.002), followed by the ileum (R²= 0.29; *P* = 0.007) and duodenum (R²= 0.18; *P* = 0.036). As the proportion of females increased, a continuous decrease in the relative weight of different intestinal segments was observed, with the decrease being more pronounced in the jejunum. Intestinal length parameters showed the highest correlation with sex composition. Total intestinal length had an R² value of 0.44 (*P* = 0.001), and duodenum (R²= 0.20; *P* = 0.028), jejunum (R²= 0.36; *P* = 0.019) and ileum (R²= 0.28; *P* = 0.037) also had relatively strong correlations. Groups with a higher proportion of males consistently had longer intestinal segments.

Table 4 shows the percentage changes in carcass characteristics (carcass weight, breast, thigh, abdominal fat) and small intestinal segments (duodenum, jejunum, ileum) in response to a change in sex ratio from 100% male (G100) to 100% female (G0). Carcass yield showed a slight increase of 0.025% in this change process. Breast weight in the G0 group decreased by 0.901% compared to the G100 group, with the largest decrease in this index being observed in the G80 group with a decrease of 5.383%. Thigh weight in the G0 group also decreased by 7.156% compared to the G100, with the largest decrease being in the G20 group (2 males+ 8 females) with a decrease of 13.114%. Abdominal fat showed a significant increase; In the G0 group, it increased by 70.135% compared to G100 group, which is the highest increase recorded in all treatments.

**Table 4:** The percentage changes in carcass characteristics (% of BW) in response to changes in male-to-female ratio.

Groups	Carcass	Breast	Thigh	Abdominal fat	Duodenum	Jejunum	Ileum
G100	0.000	0.000	0.000	0.000	0.000	0.000	0.000
G90	-0.730	-3.958	-6.702	+13.203	-8.358	-3.209	+5.945
G80	-2.332	-5.383	-5.660	+23.459	-13.800	-5.632	-1.815
G70	-2.690	-5.440	-12.588	+12.431	-13.119	-8.332	+6.665
G60	-0.973	-3.711	-10.684	+32.341	-19.356	-16.480	-11.734
G50	+0.290	-3.361	-4.763	+45.404	-19.018	-27.245	-5.452
G40	-1.151	-3.884	-4.242	+30.791	-12.987	-21.550	-19.026
G30	+0.821	+1.379	-8.802	+59.335	-16.450	-26.630	-12.658
G20	-1.660	-2.924	-13.114	+52.281	-21.962	-22.519	-27.478
G10	+0.033	-0.782	-7.102	+51.347	-27.849	-38.044	-47.519
G0	+0.025	-0.901	-7.156	+70.135	-31.638	-33.778	-30.884

Experimental groups: G100 (all males), G90 (9 males+ 1 female), G80 (8 males+ 2 females), G70 (7 males+ 3 females), G60 (6 males+ 4 females), G50 (5 males+ 5 females), G40 (4 males+ 6 females), G30 (3 males+ 7 females), G20 (2 males+ 8 females), G10 (1 male+ 9 females) and G0 (all females).

### Jejunal morphology

**Fig. 11:**
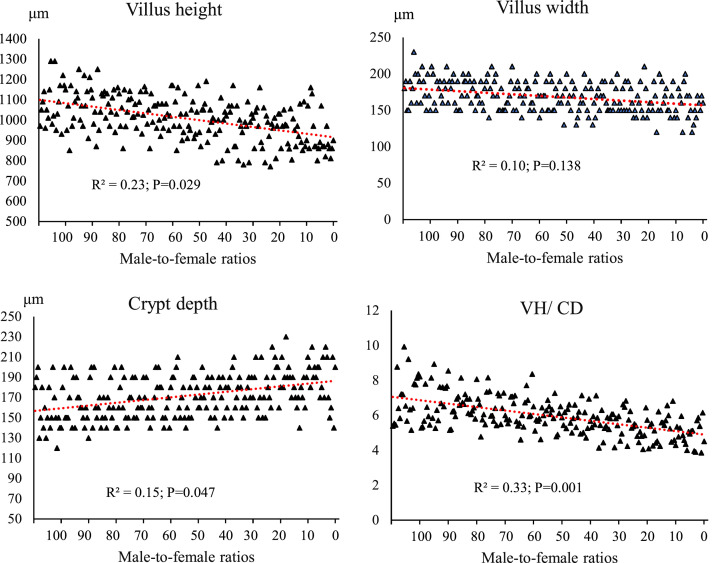
The effect of different male-to-female ratios of broiler chickens on jejunum morphology. Experimental groups: G100 (all males), G90 (9 males+ 1 female), G80 (8 males+ 2 females), G70 (7 males+ 3 females), G60 (6 males+ 4 females), G50 (5 males+ 5 females), G40 (4 males+ 6 females), G30 (3 males+ 7 females), G20 (2 males+ 8 females), G10 (1 male+ 9 females) and G0 (all females). (*n* = 20).

Fig. 11 shows the effect of gender composition on jejunal morphology. The height of the villus showed a negative and moderate correlation with changes in the sex composition of the groups (R²= 0.23; *P* = 0.029). As the proportion of female chicks increased, the height of the villus tended to decrease. The width of the villus showed a weak correlation with the sex ratio (R²= 0.10; *P* = 0.138). The depth of the crypt was negatively and relatively weakly correlated with the sex composition (R²= 0.15; *P* = 0.047), such that in the female-dominated groups, the crypt depth was greater. The ratio of VH/CD showed the highest correlation among the intestinal morphology indices (R²= 0.33; *P* = 0.001). Groups with a higher proportion of males consistently had higher values of this ratio.

**Fig. 12:**
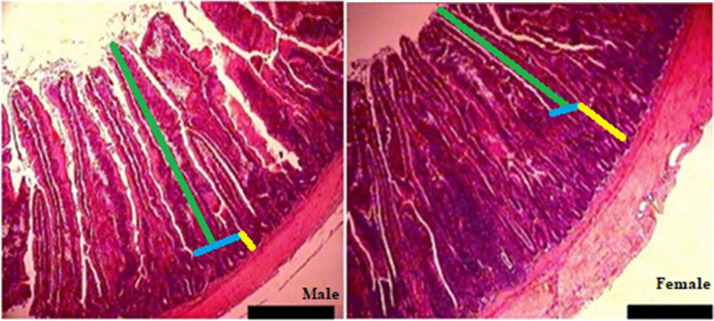
Morphological representation of the jejunum in male (left) and female (right) broiler chickens (scale bar: 100 μm). The villus height (green line) was measured from the tip of the villus to the villus-crypt junction (blue line). The crypt depth (yellow line) was measured from the villus-crypt junction to the beginning of the muscular layer.

Table 5 shows the changes in jejunal morphology indices based on the percentage difference compared to group G100 (100% male). Increasing the proportion of female broilers in the flock caused a gradual decrease in villus height; this decrease started from -1.151% in group 2 and reached -15.292% in the all-female group (G11). Crypt depth, unlike villus height and width, had an increasing trend; starting from +3.174% in group 2 and reaching the highest increase (+19.682%) in G11. The VH/CD ratio, which is an important index in assessing absorptive capacity and intestinal health, decreased significantly with increasing proportion of female broilers. Fig. 12 also shows the morphology of the jejunum of two male and female broiler chickens, with the height of the villi being significantly higher in the male.

**Table 5:** The percentage changes in jejunum morphology in response to changes in male-to-female ratio.

Groups	Villus height (μm)	Villus width (μm)	Crypt depth (μm)	VH/ CD
G100	0.000	0.000	0.000	0.000
G90	-1.151	+0.837	+3.174	-4.918
G80	-2.026	-3.072	+4.444	-7.488
G70	-3.353	-3.072	+5.714	-9.781
G60	-5.204	-6.145	+5.396	-11.066
G50	-7.738	-5.586	+8.571	-16.314
G40	-8.935	-8.938	+8.888	-17.853
G30	-10.778	-7.541	+11.428	-21.387
G20	-11.653	-8.659	+14.920	-24.013
G10	-13.274	-9.497	+16.507	-26.350
G0	-15.292	-12.290	+19.682	-30.162

Abbreviations: VH/CD: Villus height/ Crypt depth.

Experimental groups: G100 (all males), G90 (9 males+ 1 female), G80 (8 males+ 2 females), G70 (7 males+ 3 females), G60 (6 males+ 4 females), G50 (5 males+ 5 females), G40 (4 males+ 6 females), G30 (3 males+ 7 females), G20 (2 males+ 8 females), G10 (1 male+ 9 females) and G0 (all females). (*n* = 20).

### Correlation of body weight, digestibility and intestinal morphometry

**Fig. 13:**
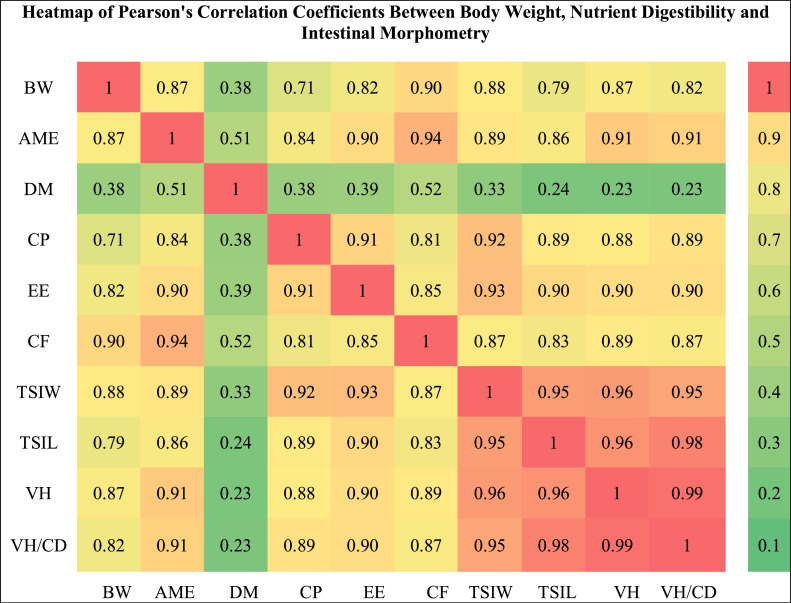
Heatmap of Pearson's correlation coefficients illustrating the relationships between body weight, nutrient digestibility parameters, and intestinal morphometric traits in broiler chickens. Abbreviations: BW: Body weight; AME: apparent metabolizable energy; DM: dry matter; CP: crude protein; EE: ether extract; CF: crude fiber; TSIW: Total small intestinal weight; TSIL: Total small intestinal length; VH: Villus height; VH/CD: Villus height/ Crypt depth.

The results of Pearson correlation analysis (Fig. 13) showed that BW was positively and strongly correlated with most of the digestibility indices and intestinal morphological characteristics. The highest correlation coefficients of BW were observed with CF (*r* = 0.90), total small intestine weight (*r* = 0.88) and villus height (*r* = 0.87). Among the digestibility indices, AME showed a very high correlation with BW (*r* = 0.87), followed by EE (*r* = 0.82) and CP (*r* = 0.71). In the section on the relationship between digestibility and intestinal morphometry, the strongest correlations between AME and CF were observed with villus height and VH/CD ratio (both *r* ≥ 0.89). In particular, total small intestine weight and total small intestine length were highly correlated with VH and VH/CD (*r* ≥ 0.95), indicating that the increase in intestinal length and weight coincided with the development of villi.

## Discussion

### Broiler performance

The results of this study demonstrated that the male-to-female ratio in broiler chickens had a direct and significant effect on performance parameters such as FI, BW, BWG, and FCR. These findings are particularly important in line with the primary objective of this research, which was to eliminate the need for sex segregation in academic trials while more accurately simulating the actual conditions of commercial poultry farms. In commercial settings, chicks are often placed as mixed sexes, and sex segregation is less common due to increased costs and management complexity. Therefore, a systematic evaluation of the effects of varying male-to-female ratios on performance can contribute to the development of more precise management and nutritional strategies. Data analysis revealed that during the starter phase (1–10 days of age), changes in sex ratio had little impact on FI, which may be attributed to the limited growth capacity at this age and the relative uniformity of metabolic requirements. However, from the grower phase (11–24 days of age), and especially during the finisher phase (25–42 days of age), an increase in the proportion of males in the flock resulted in a significant increase in feed intake. Similarly, it has been reported that female broilers have lower FI than males (Da Costa et al., 2017; England et al., 2023; Goo et al., 2019; Madilindi et al., 2018). This is likely due to the higher energy and protein requirements in males resulting from a greater genetic potential for muscle growth (Shalev & Pasternak, 1998). In addition, hormonal differences between males and females, particularly higher testosterone levels in males, can stimulate protein synthesis and elevate basal metabolic rate, thereby increasing nutrient requirements (Chen et al., 2007). The BW and BWG trends indicated that differences between treatments became more pronounced from approximately day 14, and by day 42, groups with a higher proportion of males achieved the highest BW. These results are consistent with previous reports indicating that male broilers exhibit a faster growth rate than females during the later growth stages (Da Costa et al., 2017; England et al., 2024; Madilindi et al., 2018). The mechanism underlying this difference, in addition to hormonal factors, may be related to gastrointestinal capacity; in this regard, our results showed that groups with a higher proportion of males had longer small intestines (Fig. 6) and more favorable intestinal morphology (increased villus height and decreased crypt depth) (Fig. 7, Table 5), which facilitated improved nutrient absorption and ultimately led to enhanced nutrient digestibility and AME (Fig. 3, Table 3). Madilindi et al. (2018) attributed the heavier weight for male birds to physiological differences between the sexes in feed consumption. Also, a positive genetic correlation between these traits is known (Aggrey et al., 2010). However, Zerehdaran et al. (2005) argue that the difference between males and females cannot be attributed to a single factor. Greater competition for food, aggressive male behavior, social dominance, variances in nutritional needs, and hormone effects on development and obesity are all contributing factors. In an earlier study, Henry and Burke (1998) reported that male embryos on day 20 of incubation had a higher number of myofibers than females, which may contribute to greater muscle development and ultimately result in higher final weights at the end of the rearing period. The present study showed that the difference in the performance of male and female broiler chickens starts at the grower period (Fig. 3), which is in contrast to Henry and Burke (1998). These researchers reported that sex affects the BW of broilers in the embryonic period, and male embryos are significantly heavier than females from 8 to 15 days of incubation, which is contrary to the results of the present study. In confirmation of the present study's results, Leeson and Summers (2005) reported that during the first 20 days of the rearing period, male and female broilers consume nearly the same amount of feed and exhibit similar growth. However, after this period, an increase in FI in males leads to differences in growth between the sexes. Additionally, the results of the study by Müsse et al. (2022) and Aviagen (2022) on performance indicate a similar trend, showing that sex differences in live BW increase with age.

The percentage changes calculated relative to the 100% male treatment provided further quantitative evidence of this pattern. Specifically, reducing the male proportion from 100% to 0% (all females) resulted in a 6.95% reduction in FI and a 10.99% reduction in BWG, with the latter being directly associated with a decline in CF, CP, and AME digestibility. These changes may be due to the lower gastrointestinal capacity of females to process and absorb large volumes of feed (Ravindran & Abdollahi, 2021), which aligns with our findings of a positive correlation between male proportion and digestibility indices. This indicates that the performance superiority of groups with a higher proportion of males was attributable not only to higher FI but also to greater efficiency in nutrient utilization. Moreover, the higher digestible CF levels in male groups may have enhanced intestinal peristalsis and increased the contact time between digesta and the absorptive surface, thereby improving dietary nutrient utilization (Urban et al., 2023). The set of findings shows that calculating the percentage changes in indices such as FI, BWG, and FCR relative to a reference group (e.g., 100% males) allows the researcher to express the real impact of changing the sex composition on performance numerically and comparatively. These data help in research work to create more accurate predictive models for evaluating herd efficiency and to control the effect of sex as an influential variable in testing complex hypotheses. From a practical perspective, this information allows nutritionists to simulate different management scenarios by changing the percentage of males and females and optimize economic and production decision-making.

### Coefficient of variation

The results of the present study showed that CV of FI, BWG and FCR were greater in mixed-sex pens (especially pens with more males) than single-sex male and single-sex female pens. Male broilers, although being heavier, have a larger CV, as reported by Goo et al. (2019) and Peak et al. (2000). Da Costa et al. (2017) found that CV of BW declined with age in both male and female broilers, however female broilers were more uniform than males. An increase in CV of BW can result in increased variability between repeats, affecting experiment outcomes. According to Da Costa et al. (2017), male broilers gain from mixed-sex upbringing, whereas females perform worse when raised in such an environment. These variations might have to do with competition and feeder space. Because they compete with the males for eating area, female broilers raised in mixed environments may have lower FI and weigh less. On the other hand, males raised in mixed-sex environments can readily push females and other smaller birds away from feeders, increasing FI and accelerating growth. However, compared to male birds raised in mixed-sex environments, males raised in single-sex environments would grow more slowly due to increased competition for food space. In contrast, England et al. (2022) reported that as birds become heavier, they tend to move less around the feeder. This suggests that even in mixed-sex groups, there is ample time for all birds to access the feeder, preventing males from dominating females. In the present study, it was observed that mixed-sex groups had less uniformity than single-sex male or single-sex female groups. Also, a higher level of uniformity was observed in the group with more female birds than in the group with more male birds. Therefore, probably the sense of competition and dominance in males has caused a decrease in uniformity in mixed groups.

### Nutrient digestibility and AME

In this study, the analyses clearly demonstrated that altering the male-to-female ratio in broiler chickens had significant effects on nutrient digestibility and AME. Quantifying these changes allows for better control of sex-related variation and enables more precise experimental designs. Moreover, incorporating sex ratio as a fixed factor in statistical models can reduce residual variance and increase the reliability of results. The findings indicated that shifting the composition from 100% males to 100% females resulted in a 5.34% reduction in AME, reflecting a diminished capacity for metabolizable energy production as the proportion of males decreased. Among the measured parameters, CF digestibility showed the highest sensitivity to changes in sex ratio (R²= 0.63), suggesting that alterations in gut motility and microbial community structure may play a key role in this response (Urban et al., 2023). The results of Singh et al. (2020) show that male broiler chickens had a higher digestibility coefficient for CP and organic matter compared to female broiler chickens, but the digestibility of EE, CF, calcium, and phosphorus was the same in different sexes. Ziaei et al. (2007) reported that male birds have higher nitrogen retention than female birds. These results are inconsistent, which may be due to the effect of FI on nutrient digestibility. The higher digestibility of AME, CP, EE and CF in male broilers can be attributed to differences in gut microbial population (Lee et al., 2017), gut morphology (Humer et al., 2015) and sex hormones (Zhou et al., 2018). Therefore, the researchers of the present study believe that sex hormones may increase the digestion and absorption of nutrients in male broiler chickens by affecting the intestinal microbiota and intestinal morphology. On the other hand, there is scattered evidence of gender differences in key gastrointestinal functions, such as gastrointestinal pH, pepsin levels, gastrointestinal emptying rate, and bile secretions (Lajterer et al., 2022), which requires further investigation.

Correlation analysis in the present study (Fig. 9) also revealed a very strong positive association between nutrient digestibility and both intestinal length and morphology. Increased intestinal length enhances the contact surface between digesta and absorptive cells, prolonging digesta retention time in the gastrointestinal tract and providing greater opportunity for enzymatic digestion and nutrient absorption (Tachibana et al., 2017). Notably, greater villus height increases absorptive surface area and the density of brush-border enzymes, while shallower crypt depth indicates improved cellular turnover efficiency and reduced energy costs for tissue maintenance (Chen et al., 2025). Additionally, proportional development of the submucosal and serosal muscle layers supports more effective peristalsis and uniform distribution of digesta, preventing feed stagnation or overly rapid transit and thereby optimizing digestion (Rochell et al., 2012). Furthermore, increases in the relative length and weight of the ileum and jejunum—primary sites for amino acid, fatty acid, and monosaccharide absorption—were directly associated with improved digestibility of CP, EE, and AME (Yang et al., 2013).

### Carcass traits

The results of this study indicated that altering the male-to-female ratio induced variable effects on carcass characteristics and small intestine segments. Carcass, breast, and thigh yields showed only minor variations. However, abdominal fat exhibited a substantial increase in female-dominated groups, with a 70.135% rise in the all-female group. This could be attributed to metabolic and hormonal differences between sexes, particularly in energy storage mechanisms (Ley et al., 2005). Maiorano et al. (2017) and Cygan-Szczegielniak et al. (2019) reported that the final weight, carcass weight, and breast weight of Ross chickens at the age of 42 days were higher in males than in females. In contrast, Shim et al. (2012) reported no significant effect of sex on relative breast weight. In the present study, the higher relative weight of thigh in male broilers and the higher relative weight of abdominal fat in female chickens can be partially attributed to the differences in cecal microbiota (Lumpkins et al., 2008). Cui et al. (2021) investigated the role of bacteria in the cecal contents of male and female broiler chickens, demonstrating that the cecal microbiota in male chickens is involved in various metabolic processes, including protein digestion and absorption, as well as glycan breakdown and metabolism. Abdominal fat is closely related to intramuscular fat and sensory properties of meat. As expected, female chickens had more abdominal fat than male broiler chickens, which is consistent with the findings of Van der Klein et al. (2017) and Tůmová et al. (2021). Le Bihan-Duval et al. (1998) reported that more abdominal fat in female broilers could be due to differences in hormone production between males and females and their effect on fat deposition.

Small intestine characteristics were more sensitive to sex composition. The most pronounced changes were observed in relative jejunal weight and total intestinal length, with male-dominated groups exhibiting greater length and weight in various intestinal segments. This phenomenon can be explained by several physiological and metabolic factors. First, male broilers have higher growth rates and energy requirements, necessitating greater digestive capacity, which promotes the development of intestinal tissues—especially the jejunum and ileum (Stęczny & Kokoszyński, 2020). Second, hormonal differences, particularly elevated testosterone levels in males, stimulate anabolic processes and enhance epithelial cell proliferation in the intestine (West & Phillips, 2010). Furthermore, studies have shown that males have higher feed intake and faster digesta passage rates, which increase the mucosal surface area and consequently lead to longer and heavier intestines (Goo et al., 2019). In addition, genetic differences in muscle growth patterns and supportive tissue development result in a more developed digestive system in males to sustain their higher body mass (Biesek et al., 2022).

### Jejunum morphology

A progressive decrease in villus height with an increasing proportion of females suggests that a predominance of males may help maintain taller villus structures, thereby providing greater absorptive surface area. Higher villus height is generally associated with an increased epithelial contact surface and improved nutrient absorption (Wang et al., 2025). This phenomenon may be attributed to hormonal differences between sexes, particularly the role of testosterone in stimulating epithelial cell proliferation and increasing villus length (Mfoundou et al., 2022). Consistent with the present findings, Humer et al. (2015) reported greater villus height in male broilers compared with females. Similarly, Tomaszewska et al. (2018), comparing the effects of diets containing Saccharomyces cerevisiae in Japanese quail, observed higher villus height and absorptive surface area in males than females. Such sex-based differences in villus height can be related to intestinal weight and length as well as microbial composition. In the present study, villus height was strongly and positively correlated with intestinal weight and length, and higher BW was associated with longer and heavier intestines and taller villi. Furthermore, male broilers have been reported to harbor greater numbers of *Lactobacillus* spp., known for their ability to degrade simple carbohydrates and oligosaccharides (Apajalahti & Vienola, 2016). More efficient oligosaccharide breakdown can reduce intestinal viscosity and increase villus height (Barasch & Grimes, 2021). In addition, England et al. (2024) reported that the cecal content of male broilers contained a higher relative abundance of *Faecalibacterium spp*., which influence the production of short-chain fatty acids (Bjerrum et al., 2006). Short-chain fatty acids produced by *Faecalibacterium* may stimulate epithelial cell proliferation in the intestine, resulting in taller villi and greater absorptive surface area (Tomaszewska et al., 2018).

### Correlation of body weight, digestibility and intestinal morphometry

The correlation results in this study revealed that BW exhibited a very strong and positive association with most indices of nutrient digestibility and intestinal morphology, particularly with AME, total small intestine weight, and villus height. Biologically, this indicates that heavier birds generally possess larger intestines and taller villi, potentially increasing the surface area available for nutrient contact and thereby improving absorption efficiency (Hollemans et al., 2020; Kyoung et al., 2023). However, a critical consideration in interpreting these findings is that intestinal morphology studies often report absolute values of villus height or crypt depth without adjustment for BW (Waghmare et al., 2025). Since experimental birds do not necessarily have identical BWs, differences in BW can artificially inflate or reduce these morphological indices. In such cases, greater BW may mechanically result in larger absolute dimensions of the intestine and villi, and failure to adjust for BW can lead to experimental bias and misinterpretation of treatment effects. For instance, if birds from one treatment group are heavier, the observed increase in villus height might merely reflect larger body size rather than a genuine physiological adaptation to the treatment. To obtain more accurate results, it is recommended that intestinal morphological indices be expressed in relative terms (e.g., per unit BW or relative to total intestinal length/weight) or that data be statistically adjusted using covariate models such as ANCOVA to disentangle the true treatment effect from body size effects. This adjustment is particularly crucial in nutritional, genetic, and sex-comparison studies, where BW differences between treatments are common. Failure to perform such corrections may lead to erroneous conclusions regarding mechanisms of nutrient absorption and intestinal development. Also, given the strong positive correlation between nutrient digestibility and BW observed in this study, this consideration is equally critical in the design and interpretation of digestibility trials. When heavier birds are selected within a treatment group for digestibility assessments, they are likely to exhibit higher digestibility values, even if the improvement is unrelated to the diet or additive being tested. In such cases, the apparent enhancement in digestibility may be mistakenly attributed to the treatment, whereas the primary factor is actually BW differences among birds. Therefore, in digestibility studies, it is essential to either statistically adjust for BW or control it through the selection of weight-matched samples, thereby ensuring greater accuracy and validity in result interpretation.

## Conclusions

The findings demonstrated that altering the male-to-female ratio induced clear and quantifiable changes in body weight, nutrient digestibility, and intestinal morphological traits, with distinct and statistically meaningful correlation patterns across growth phases. Quantifying these percentage changes provides a robust basis for estimating sex ratio effects and facilitates the development of predictive approaches that improve the alignment between academic research and commercial broiler production without the need for complete sex segregation. Given that the present study was conducted using the Ross 308 broiler strain, it is recommended that future studies evaluate the magnitude and pattern of sex ratio–related changes across different genetic strains to further validate and generalize these findings.

## Animal ethics

All the methods applied in the present study were approved by the Animal Ethics Committee of the Animal Science Faculty at Urmia University, under protocol number IR-UU-AEC-3.76.

## CRediT authorship contribution statement

**Motaleb Ebrahimi:** Writing – original draft, Project administration, Methodology, Investigation, Funding acquisition, Formal analysis. **Mohsen Daneshyar:** Writing – review & editing, Writing – original draft, Visualization, Validation, Supervision, Software, Resources, Project administration, Methodology, Investigation. **Sina Payvastegan:** Writing – review & editing, Writing – original draft, Visualization, Validation, Supervision, Software. **Hamed Ahmadi:** Writing – review & editing, Visualization, Validation, Software, Methodology, Formal analysis, Data curation, Conceptualization.

## Declaration of competing interest

The authors declare that they have no conflicting of interests
